# The relationship between non-suicidal self-injury and borderline personality disorder symptoms in a college sample

**DOI:** 10.1186/2051-6673-1-14

**Published:** 2014-09-25

**Authors:** Lauren J Brickman, Brooke A Ammerman, Amy E Look, Mitchell E Berman, Michael S McCloskey

**Affiliations:** Department of Psychology, Temple University, 1701 N. 13th Street, Weiss Hall, Philadelphia, PA 19122 USA; Department of Psychology, Lee Blvd, Mississippi State University, Mississippi, MS 39759 USA

**Keywords:** Borderline personality disorder, BPD, BPD symptoms, Self-harm, Non-suicidal self-injury

## Abstract

**Background:**

Non-suicidal self-injury (NSSI) is a major concern in both clinical and non-clinical populations. It has been approximated that 65-80% of individuals with borderline personality disorder (BPD) engage in some form of NSSI. Despite such high co-morbidity, much still remains unknown about the relationship between NSSI and BPD symptomatology. The goal of the current study was to identify individual BPD symptoms and higher order BPD factors that increase one’s vulnerability of NSSI engagement among a college sample. It was hypothesized that the BPD factor of emotion dysregulation and the BPD symptoms of affect instability and intense anger/aggression would be associated with the presence and frequency of NSSI.

**Method:**

Seven hundred twenty four undergraduates (61.2% female) completed self-report measures of BPD symptomology and NSSI history.

**Results:**

Regression analyses revealed that among the individual BPD symptoms, past suicidality, impulsivity, chronic emptiness, and identity disturbance were each significantly, positively associated with lifetime history of NSSI, whereas unstable relationships were negatively associated with lifetime history of NSSI. The BPD symptom associated with NSSI frequency was dissociation. Among the BPD factors, emotion dysregulation and disturbed relatedness were both associated with NSSI history, but only disturbed relatedness was associated with NSSI frequency.

**Conclusion:**

Findings show partial support for the importance of emotion dysregulation in the relationship between NSSI and BPD symptomatology, but also suggest that the relationship may be more complex and not solely based on emotion dysregulation.

## Background

Non-suicidal self-injury (NSSI), defined as self-directed and intentional behavior that causes harm or destruction to bodily tissue without the intent to die [1, 2], is a prevalent and significant public health concern [3, 4]. It is associated with psychological distress in addition to physical damage that, at the extreme, may require medical attention [5]. Originally thought to be relatively rare and limited to psychiatric populations, NSSI is now understood to occur frequently in both clinical and non-clinical populations: approximately 4% to 6% of the general adult population reports engaging in NSSI [6, 7]. Further, this behavior seems to occur at higher rates among adolescents and young adults with 15% to 38% of college students reporting to engage in NSSI [8, 9]. Associations have been suggested between NSSI and many psychiatric conditions; however, borderline personality disorder may have a unique and robust relation with NSSI [10, 11].

Borderline personality disorder (BPD) is a serious psychiatric condition characterized by affect dysregulation, instability in interpersonal relationships and self-image, and self-harm [12]. Recent epidemiological studies have found it to occur in approximately 2%-6% of the general population [13, 14], and even higher estimates in clinical samples with 11% outpatient and 19% of inpatient samples being diagnosed with BPD [15]. BPD is associated with many negative outcomes, including self-harm across the spectrum of lethality [16]. As many as 9% of individuals with BPD die by suicide [17] and approximately 65-80% of individuals engage in NSSI [11, 18]. Though researchers have identified a strong association between NSSI and BPD [16, 19], much less is known about the association of specific BPD symptoms with NSSI engagement.

The heterogeneity of BPD has long been an issue. Researchers have attempted to identify homogeneous subsets of BPD symptoms to identify subtypes of BPD and to more parsimoniously examine the relationships between BPD symptomatology and putatively associated factors. Theoretical models have proposed five dimensions [19] or subtypes [20] of BPD, however, more recent empirical studies (e.g., [21, 22]) have largely supported a three factor model of BPD symptomatology (but also see [23]) consisting of behavioral dysregulation, disturbed relatedness, and emotion dysregulation, which are thought to represent core dimensions of borderline personality. Behavioral dysregulation refers specifically to self-harm (i.e., NSSI and suicidal behavior) and impulsivity, while disturbed relatedness reflects a disturbed sense of self and relatedness to others. Despite a lack of direct investigation, the disturbed relatedness and emotion dysregulation factors have been found to indirectly relate to NSSI. With respect to disturbed relatedness (i.e., paranoid ideation, emptiness, identity disturbance, and unstable relationships), an interpersonal function of NSSI has been identified and suggests the behavior may be utilized as a way to communicate with [24] or to elicit affection or attention from a loved one [5]. Individuals who engage in NSSI also report engaging in the behavior to reduce feelings of emptiness [22] and NSSI is associated with reports of greater levels of depersonalization and drug-free hallucinations or delusions [11]. However, it is the emotion dysregulation factor (i.e., anger, affective instability and frantic efforts to avoid abandonment) that is most strongly associated with NSSI.

Emotion dysregulation, which entails the inability to effectively regulate one’s inner emotional experience, such as the emotions an individual experiences, when the emotions are experienced, and the resultant behavior or expression [19, 25–27], is thought to be a core deficit in BPD [28, 29]. Indeed, approximately half of individuals with BPD endorse affect lability and/or problems with anger and aggression, with fear of abandonment less common, but highly predictive of BPD [30]. Emotion dysregulation is also associated with NSSI [31, 32], with the large majority of NSSI acts serving an emotion regulation function [2]. This is particularly true for individuals with BPD, as over 95% of women with BPD who engage in NSSI report doing so for (among other things) emotional relief [2, 31]. Furthermore, in clinical samples affect lability and anger problems discriminate those who do and do not engage in self-harm [33, 34]. Thus, in clinical samples of individuals with BPD, research shows emotion regulation is associated with NSSI.

Despite our knowledge of the association between BPD and NSSI, the extent to which these symptoms are independently associated with NSSI in a general college sample remains unclear. The goal of the current study was to examine the relationship between BPD symptomatology and NSSI in a general college sample by examining both individual BPD symptoms and the three factors they comprise. Given several BPD criteria have been found to be associated with NSSI [35–37], it was expected all BPD symptoms and factors would discriminate college students with and without NSSI. However, when all symptoms or factors were examined simultaneously, the factor of emotion dysregulation and specifically the symptoms of affect instability and anger/aggression were hypothesized to be independently associated with both the likelihood of engaging in NSSI and the frequency of NSSI among those who engage in the behavior. Consistent with past research [38], a history of suicidality was also hypothesized to independently differentiate those with and without a history of NSSI.

## Methods

### Participants

Participants were 788 male and female undergraduate students from a large urban university taking part in a larger study. After all participants completed study measures, two groups were formed to clearly discriminate between participants: those with a history of NSSI (NSSI+) and without a history of NSSI (NSSI-). Those in the NSSI + group reported three or more lifetime episodes of NSSI (*n* = 136), whereas those in the NSSI- group reported no lifetime NSSI (*n* = 588). Individuals with repeated self-injury have been identified as distinctly different from those who have engaged in the behavior non-repetitively, representing a more severe or impaired class [39–41]. To best capture individuals with repeated self-injury, 64 participants were removed from analyses because they reported 1 or 2 lifetime episodes of NSSI. The final sample consisted of 724 participants (281 males, 443 females), aged 17 to 57 (*M* = 21.23, *SD* = 3.87) who were predominately Caucasian (59.30%), African American (14.8%), and Asian (12.6%).

### Materials

#### Self-injury

The Forms and Function of Self-Injury Scale (Jenkins A, Connor B, McCloskey, MS, Alloy, LA: The Form and Function of Self-Injury Scale (FAFSI): The development and psychometric evaluation, submitted) is a multi-part self-report measure used to determine lifetime history of NSSI and to provide an estimate of the frequency of NSSI behavior (across NSSI categories) engaged in by each participant. For this study, only the first section of the measure assessing the frequency and methods of NSSI was utilized. Participants were asked, “Have you ever, intentionally or on purpose, hurt yourself in the following ways, without the intention of killing yourself?” Then, for each of 13 possible types of NSSI (e.g., self-cutting, self-burning, self-hitting) they reported the number of times they engaged in each listed behavior (e.g., “cut yourself, either to cause pain or draw blood”). The internal consistency (KR-20 = .83 for NSSI behaviors) and factor structure of the FAFSI has been supported (Jenkins A, Connor B, McCloskey, MS, Alloy, LA: The Form and Function of Self-Injury Scale (FAFSI): The development and psychometric evaluation, submitted). In the present study the internal consistency of the NSSI behaviors was also strong (KR-20 = .81).

#### Borderline personality symptomology

The McLean Screening Instrument for Borderline Personality Disorder (MSI-BPD [39]), a 10-item self-report measure of BPD features, was used to assess BPD symptoms. Convergent and concurrent validity of the measure have been supported [40]. In the current study the MSI-BPD demonstrated good reliability (α = .83). Further, when compared with a validated structured interview of BPD diagnosis, both sensitivity and specificity of the MSI-BPD were above .90 [37]. The current study used both individual MSI-BPD items (i.e., BPD symptoms) and BPD factors. Composition of the BPD factors was based on the three-factor model by Sanislow et al. [41] and supported by more recent confirmatory factor analyses [21, 22]. These factors consisted of: 1. emotion dysregulation (“been extremely moody”; “felt very angry a lot of the time”; “made desperate efforts to avoid feeling abandoned or being abandoned”); 2. disturbed relatedness (“closest relationships troubled by a lot of arguments or repeated breakups”; “felt you had no idea who you are or that you have no identity”; “chronically felt empty”; “been distrustful of other people”; “frequently felt unreal or as if things around you were unreal”); and 3. behavioral dysregulation (“deliberately hurt yourself physically/made a suicide attempt”; “at least two other problems with impulsivity”). Factor scores were derived by totaling composite items. In the current sample the MSI-BPD showed good internal consistency (KR-20 = .83).

### Procedures

Participants were recruited through an online research participation system at a large urban university, and were enrolled at the university at the time of the study. They completed a series of self-report measures as part of a larger study on aggression and self-aggression through a secure website as approved by the University’s Institutional Review Board. All participants provided informed consent and received course credit for their participation. Participants were excluded from the study if they failed to complete the measures of interest for the current study, which were questionnaires assessing self-injurious behavior and borderline personality disorder symptomatology.

## Results

The factor of behavior dysregulation was not included in the primary regression analyses as it includes acts of parasuicidal behavior (i.e., NSSI) and the goal of the current study was to identify risk factors beyond the self-injury criterion itself. The borderline personality disorder screening instrument used in this study (MSI-BPD) differentiates suicide attempts/gestures from NSSI; however, given the suggested relationship between NSSI and the other components of the behavioral dysregulation factor, i.e., suicidal behavior and impulsivity [38], impulsivity and suicidal behavior were included in the analyses of individual BPD symptoms.

### Participant characteristics

NSSI + and NSSI- groups were compared on the demographic variables of gender, race and age. The NSSI + participants were more likely to be female, χ^2^ (1, *N* = 723) = 11.22, *p* < .001. There were also significant group differences on race, χ^2^ (1, *N* = 724) = 5.43, *p* < .05, with post hoc single DF χ^2^ analyses revealing NSSI + participants were significantly more likely to be Caucasian than African American or Asian (both *p* < .05) relative to NSSI- participants. No significant differences between NSSI + and NSSI- participants with regard to proportion of African American, Asian, or other were found. Consequently, race was dichotomized into Caucasian and minority (African Americans, Asians, and others) and controlled for in primary analyses. No differences between NSSI + and NSSI- participants existed with respect to age, *t*(722) = .24, *p* > .05. With regard to individual BPD symptoms, NSSI + participants were more likely to endorse each criterion (χ^2^ (1, *N* = 724) = 4.47 – 94.31; *p* < .001 to .04) and higher levels on each subdomain (*t*(722) = 8.65 – 26.05, all *p* < .001) than NSSI- participants. See Table 1 for descriptive information. Additionally, NSSI + individuals (*M* = 6.07, *SD* = 2.82) endorsed an overall greater number of BPD symptoms than NSSI- participants (*M* = 2.20, *SD* = 2.37), *t*(722) = 16.56, *p* < .001. Of the overall sample, 92 participants (13.12%), endorsed 7 or more items on the MSI-BPD, which is the suggested cutoff for a BPD diagnosis [37]. The difference in proportion of NSSI + (37.5%, N =51) and NSSI- (7.0%, N = 41) above this cutoff was significant, χ^2^ (1, *N* = 724) = 103.81, *p* < .001. Among NSSI + participants, the number of NSSI episodes ranged from 3–4894, with a mean of 112.75 (SD = 476.05) and a median number of 21. The most common type of self–injury was cutting (71.20%), followed by banging head (37.30%), pinching self (32.20%), and scratching or scraping skin (30.50%).

**Table 1:** **Endorsement of MSI-BPD items and factor score as a function of NSSI group**

MSI-BPD	Overall sample (***N*** = 724)	NSSI + group (***N*** = 136)	NSSI – group (***N*** = 588)
Factor score *M* (SD)			
Emotion Dys**	0.85 (1.02)	1.58 (1.03)	0.69 (0.94)
Disturbed relatedness**	1.49 (1.53)	2.57 (1.66)	1.24 (1.38)
Behavioral Dys**	0.57 (0.83)	1.91 (0.70)	0.26 (0.47)
Items endorsed% (N)			
Relationship instability*	38.4% (N = 278)	46.3% (N = 63)	36.6% (N = 215)
Impulsivity**	30.0% (N = 217)	59.6% (N = 81)	23.1% (N = 136)
Unstable affect**	38.4% (N = 278)	67.6% (N = 92)	31.6% (N = 186)
Angry**	26.7% (N = 193)	47.1% (N = 64)	21.9% (N = 129)
Distrustful of others**	40.7% (N = 295)	64.7% (N = 88)	35.2% (N = 207)
Felt unreal**	23.1% (N = 167)	39.7% (N = 54)	19.2% (N = 113)
Chronic emptiness**	25.4% (N = 184)	58.1% (N = 79)	17.9% (N = 105)
Identity**	21.8% (N = 158)	48.5% (N = 66)	15.6% (N = 92)
Avoid abandonment**	20.9% (N = 151)	44.1% (N = 60)	15.5% (N = 91)
Suicidality**	21.1% (N = 153)	100% (N = 136)	2.9% (N = 17)

*Note:* *p < .05, **p < .001; MSI-BPD, McLean Screening Instrument for Borderline Personality Disorder, NSSI = Non-Suicidal Self-Injury, Dys = Dysregulation.

### NSSI status

Individual BPD criteria, as measured by the MSI-BPD, were assessed for association with engagement in NSSI via a hierarchical logistic regression, in which the control variables of gender and race were entered in the first step and the MSI–BPD items were entered in the second step. The first step consisting of race and gender was significant χ^2^ (10, *N* = 724) = 20.14, *p* < .001 (Cox & Snell *R*^2^ = 03%; Nagelkerke *R*^2^ = 04%). The second step consisting of MSI-BPD items also significantly distinguished NSSI + and NSSI- participants, χ^2^ (10, *N* = 724) = 175.43, *p* < .001 (Cox & Snell *R*^2^ = 23%; Nagelkerke *R*^2^ = 38%). Among the individual items, history of suicide attempts, impulsivity, chronic feelings of emptiness, and identity disturbance were all independently, positively associated with NSSI + status while unstable relationships was negatively associated with NSSI + status (see Table 2).

**Table 2:** **Hierarchical logistic regression of individual borderline symptoms on presence of non-suicidal self-injury**

Variable	***B***	***SE B***	Wald	OR	95% CI for OR
Step 1					
Gender	0.67*	.26	6.55	1.94	1.17-3.23
Race	−0.67**	.25	6.96	0.52	0.32-0.84
Step 2					
Unstable relationships	−0.76**	.27	7.89	0.47	0.28-0.80
Suicide attempt	2.10***	.36	34.60	8.19	4.06-16.49
Impulsivity	0.92***	.26	12.92	2.51	1.52-4.13
Affective lability	0.34	.28	1.42	1.40	0.81-2.44
Inappropriate/intense anger	0.22	.28	0.63	1.25	0.72-2.16
Stress related paranoia	0.41	.26	2.50	1.51	0.91-2.53
Severe dissociation	−0.43	.30	2.07	0.65	0.36-1.17
Emptiness	0.94**	.28	11.12	2.56	1.47-4.45
Identity disturbance	0.72**	.27	6.91	2.05	1.20-3.50
Fear of abandonment	0.29	.29	1.03	1.33	0.77-2.31

*Note:* *p < .05, **p < .01, ***p < .001; OR = Odds Ratio; CI = Confidence Interval.

A second hierarchical logistic regression assessed the extent to which the emotion dysregulation and disturbed relatedness factors were associated with NSSI group membership. Again the first step consisting of race and gender was significant at the same level as before (see above). The second step, consisting of the two BPD factors, significantly distinguished NSSI + and NSSI- participants, χ^2^ (2, *N* = 724) = 95.94, *p* < .001 (Cox & Snell *R*^2^ = 15%; Nagelkerke *R*^2^ = 24%). Both emotion dysregulation [*B (SE)* = .44 (.12), Wald = 13.44, *p* < .001, OR = 1.55, 95% CI = 1.23 – 1.97] and disturbed relatedness [*B (SE)* = .39 (.08), Wald = 22.55, *p* < .001, OR = 1.47, 95% CI = 1.26 – 1.73] were positively associated with NSSI + status.

### NSSI frequency

A pair of hierarchical (step 1 = race and gender, step 2 = predictors) linear regressions were used to assess which criteria and factors were most strongly associated with NSSI frequency among individuals in the NSSI + group. In the hierarchical linear regression assessing individual BPD symptoms, the results indicated the model as a whole was not significantly associated with NSSI frequency (*R*^2^ = .09, *F* (12,105) = .84, *p* = .61). Among the individual BPD symptoms only severe dissociation was significantly related to NSSI frequency (see Table 3). In the hierarchical regression assessing BPD factors, the first step associated with race and gender was not significant, *R*^2^ = .01, *F* (2,133) < 1. However, the second step assessing the two BPD factors was significant (Δ *R*^2^ = .05, Δ *F* (2,131) = 3.55, *p* = .03). Specifically, the disturbed relatedness factor [*B (SE)* = 30.42 (11.48), *Beta* = .28, *t* = 2.65, *p* = .009] was associated with increased frequency of NSSI, Surprisingly, emotion dysregulation was not associated with NSSI frequency [*B (SE)* = −22.78 (18.71), *Beta* = −.12, *t* = −1.21, *p* = .23].

**Table 3:** **Hierarchical linear regression model of individual borderline symptoms on frequency of non-suicidal self-injury**

Variable	b	***SE B***	β	***R*** ^2^	Δ***R*** ^2^	***F*** for Δ***R*** ^***2***^
Step 1				.01	.01	.78 (2, 133)
Gender	17.10	40.58	.04			
Race	28.21	37.43	.07			
Step 2				.08	.07	.94 (10, 123)
Unstable relationships	22.00	37.60	.06			
Suicide attempt	−8.76	38.09	-.02			
Impulsivity	14.34	35.64	.04			
Affective lability	5.28	42.46	.01			
Inappropriate/intense anger	−25.75	37.07	.07			
Stress related paranoia	28.17	39.18	.07			
Severe dissociation	81.52	40.45	.22*			
Emptiness	9.64	41.74	.03			
Identity disturbance	3.92	39.49	.01			
Fear of abandonment	−41.54	38.85	−.11			

*Note:* *p < .05

## Discussion

Though the link between NSSI and BPD pathology has been well established [39], this study was the first to examine the BPD factors as well as individual symptoms in relation to NSSI in a college population. It was hypothesized that the BPD factor of emotion dysregulation and two of its component symptoms, affective instability and anger/aggression, would be associated with NSSI group membership as well as the frequency of NSSI. It was also hypothesized that suicidality would predict NSSI status. The findings of the current study provide only partial support for these hypotheses. The emotion regulation factor did predict NSSI group membership, as did endorsement of suicidality. However, none of the symptoms that comprise the emotion regulation factor were independently associated with either NSSI status or frequency. The disturbed relatedness BPD factor and some of the BPD symptoms that comprise that factor were also independently associated with NSSI status and frequency. Overall, the findings suggest that an array of BPD symptoms and factors, apart from those most directly associated with ability to modulate affect, may play a significant role in vulnerability for engagement in NSSI.

Of specific interest in the current study is the support for the association between NSSI and emotion dysregulation. Individuals in the NSSI + group, as compared to the NSSI- group, reported higher levels on the BPD factor of emotion dysregulation and subordinate BPD symptoms of affect lability, fear of abandonment and anger/aggression. Further, the emotion dysregulation factor discriminated between those in NSSI + and NSSI- group. This is consistent with previous NSSI research in BPD and other samples [31, 42–44] suggesting difficulty in effectively modulating one’s own emotions may significantly confer risk for self-injurious behavior. Despite the relationship between the emotion dysregulation factor and history of NSSI, neither affect lability nor intense anger/aggression was uniquely related to NSSI behavior. This was somewhat surprising considering the overall relationship between emotion regulation and NSSI, as well as past studies showing emotion dysregulation is associated with self-harm in BPD [45]. However, unlike past studies, the current study utilized a non-clinical sample of which only a relatively small percentage (13%) of individuals reported meeting the diagnostic cutoff for BPD (based on the MSI-BPD screening measure), as compared to a clinical sample where all participants were seeking treatment for BPD. Thus, it may be that among individuals with milder forms of emotion dysregulation than is typically seen in BPD, only when affective lability is compounded by chronic intense anger or aggression and/or fear of abandonment, is the likelihood to engage in NSSI significantly enhanced. This would be consistent with research showing that among individuals with high negative affect (i.e., major depression), trait aggression was key in predicting who would engage in suicidal self-harm [33].

The disturbed relatedness factor and several of the BPD symptoms comprising this factor were independently associated with NSSI status. Within the disturbed relatedness factor, endorsement of the BPD symptoms that reflect intrapersonal difficulties (i.e., chronic emptiness and identity disturbance) independently distinguished NSSI+; whereas, endorsement of the most definitively interpersonal (turbulent relationships) symptom was associated with NSSI–status. In line with these results, feelings of emptiness is an antecedent and motivation for engaging in NSSI among young adults [46], and identity confusion has been associated with a history of NSSI among adolescents [47]. Present findings suggest that these intrapersonal factors may be particularly salient to the initiation of NSSI in a college population, and thus may represent a potential target for NSSI prevention in this population.

As stated, endorsement of the unstable relationships criterion was not associated with NSSI non-membership, which is inconsistent with previous research by Muehlenkamp and colleagues [48] who found unstable interpersonal relationships were associated with both NSSI and suicide attempts. This study was conducted among an adolescent outpatient clinical sample, however. Though it is not clear, one possible explanation for the discordant finding is that in general college samples turbulent relationships are more normative and that alienation, rather than interpersonal distress, is more closely linked with NSSI.

As hypothesized, a history of suicidality was independently associated with NSSI status. Several previous studies have shown a close relationship between NSSI and suicidality (e.g., [49, 50]), and the present findings replicate and extend this in a diverse college sample. Furthermore, our finding that impulsivity also discriminated between NSSI + and NSSI- participants provides support to a mixed literature on the relationship between impulsivity and NSSI [36, 37].

The disturbed relatedness factor, and specifically the symptom of severe dissociation, was also independently associated with NSSI frequency among those endorsing a history of NSSI. However, it should be noted that despite being statistically significant, these effects were relatively small. In the context of BPD symptomatology it may be the case that dissociation and subsequent NSSI is serving to repeatedly reduce awareness of intolerable, intense negative emotions. The relationship found between dissociation and NSSI is consistent with previous research suggesting that dissociative symptoms of derealization, depersonalization, and psychogenic amnesia are found to commonly precede the urge to engage in NSSI [51]. It is thought that self-injury may influence dissociative symptoms through affect modulation (e.g., regaining a sense of reality) or to stop feeling empty, which is another common characteristic among those with BPD.

Several limitations of the study should be addressed. First, the study relied primarily on participant report, which may be prone to non-disclosure and difficulty in memory recall. The latter is particularly important in the current study as the nature of BPD symptomatology can be complex and particularly difficult to identify. Data were combined in a manner that did not take different forms and function of NSSI into account, instead investigating the overall construct. This is noteworthy as recent findings suggest that in some cases NSSI function may moderate the relationship between BPD symptomology and NSSI behavior [45]. Finally, the study employed a cross sectional design to describe the relationship between BPD symptomatology and NSSI, prohibiting conclusions to be drawn on the extent of BPD symptomatology as risk factors for NSSI.

Notwithstanding such limitations, there are strengths to the current study that help provide direct implications for clinical practice. Results highlight certain BPD symptoms that may be helpful in identifying individuals who could be at risk for NSSI engagement, regardless of BPD diagnostic status. This may be particularly useful in a university counseling center setting, especially given the current sample. For example, individuals presenting with chronic feelings of emptiness or difficulties in identity formation, or reporting a combination of unstable mood, angry temperament and abandonment concerns, may be targets for intervention as they are potentially at increased likelihood of engaging in NSSI. Undergraduate student samples are often considered a limitation to generalizability; however, there are high rates of NSSI among teens and young adults, in addition to the prevalence of onset being during this time period, making it a particularly important age range to target intervention and prevention efforts.

## Conclusion

Despite these limitations, the present findings represent a useful contribution to understanding self-injury in the context of BPD. As many self-injuring individuals never seek out mental health treatment [42], there is a strong need to identify correlates and potential risk factors of the behavior. The current findings suggest that the assessment of BPD symptomology as individual criterion or as subdomain may help further identify those at risk for self-injury.
